# The case of a 69‐year‐old man with COVID‐19 and encephalopathy

**DOI:** 10.1002/acn3.51250

**Published:** 2020-11-17

**Authors:** Trevor E. Cline, Navdeep Sangha

**Affiliations:** ^1^ Kaiser Permanente Los Angeles Medical Center Los Angeles CA USA

## Abstract

A 69‐year‐old man with uncontrolled type 2 diabetes presented to an outside hospital with altered mental status. He progressed from being argumentative to encephalopathic and agitated by the evening with urinary frequency, urinary urgency, nausea, and vomiting. His vital signs were normal, and he had no focal neurological deficits on presentation. He was generally encephalopathic, only groaning with no ability to follow commands. He was found to have diabetic ketoacidosis on initial labs. A left parietal hypodensity on CT Head was found, and he was positive for Sars‐COV‐2.

## Diagnosis

**Figure 1:**
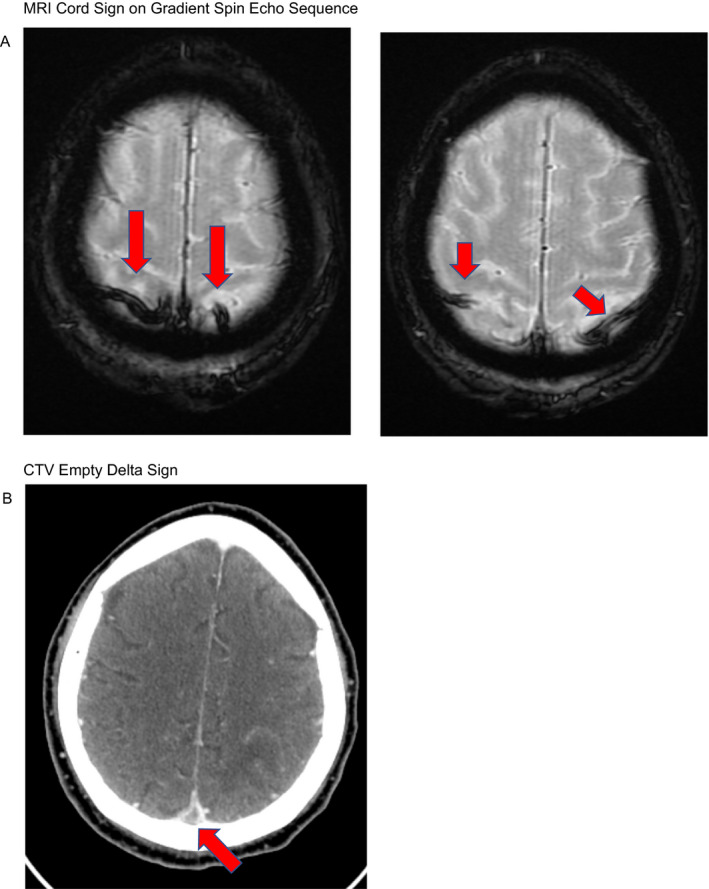
(A) MRI Cord Sign on Gradient Spin Echo Sequence and (B) CTV Empty Delta Sign.

Cerebral venous sinus and cortical vein thrombosis associated with COVID‐19 (Figure 1).^1^

## Take‐Home Points

- COVID‐19 disease is associated with a hypercoagulable, hyperinflammatory state,^2^ and any change in neurological status in conjunction with any suspicion of COVID disease should trigger neurological consultation and urgent neuro imaging with a non‐contrast CT Head.
- Cerebral venous thrombosis is an uncommon sub type of stroke that has non‐specific initial symptoms but is associated with infection and hyper‐coagulable states. Characteristic signs include a “empty delta” defect in filling on a contrast enhanced CT Venogram of the head as well as a “cord sign” on a gradient spin echo sequence on a non‐contrast MRI of the head.^3^, ^4^
- Standard of care is empiric treatment with unfractionated heparin or low molecular weight heparin as soon as CVST is diagnosed.^2^
